# Functional Capacity Among Brazilian Older Adults 12 Months After COVID-19 Infection: A Cross-Sectional Study

**DOI:** 10.3390/jcm14010009

**Published:** 2024-12-24

**Authors:** Flávia Cristina Sierra de Souza, Carlos Laranjeira, Maria Aparecida Salci, Carla Franciele Höring, Herbert Leopoldo de Freitas Góes, Vanessa Denardi Antoniassi Baldissera, Débora Moura, Viviani Camboin Meireles, Maria Fernanda Prado, Susanne Elero Betiolli, Jesús Puente Alcaraz, Carlos Alexandre Molena Fernandes, Lígia Carreira

**Affiliations:** 1 Department of Postgraduate Nursing, State University of Maringá, Avenida Colombo, 5790-Campus Universitário, Maringá 87020-900, Brazil; flavia.souza@unespar.edu.br (F.C.S.d.S.); masalci@uem.br (M.A.S.); hlfgoes@uem.br (H.L.d.F.G.); vanessadenardi@hotmail.com (V.D.A.B.); dromoura2@uem.br (D.M.); vcmeireles@uem.br (V.C.M.); mfpprado@gmail.com (M.F.P.); carlosmolena126@gmail.com (C.A.M.F.); lcarreira@uem.br (L.C.); 2 School of Health Sciences, Campus 2, Polytechnic University of Leiria, Morro do Lena, Alto do Vieiro, Apartado 4137, 2411-901 Leiria, Portugal; 3 Centre for Innovative Care and Health Technology (ciTechCare), Polytechnic University of Leiria, Campus 5, Rua das Olhalvas, 2414-016 Leiria, Portugal; 4 Comprehensive Health Research Centre (CHRC), University of Évora, 7000-801 Évora, Portugal; 5 Departamento de Estatística, State University of Maringá, Avenida Colombo, 5790-Campus Universitário, Maringá 87020-900, Brazil; cfhoring2@uem.br; 6 Programa de Pós-Graduação em Enfermagem, Universidade Federal do Paraná, Curitiba 80060-000, Brazil; susanne@ufpr.br; 7 Department of Nursing, University of Burgos, 09001 Burgos, Spain; jpalcaraz@ubu.es

**Keywords:** functional status, COVID-19, daily living activities, older people, Brazil

## Abstract

**Background/Objectives:** Evidence suggests that older adults who survived COVID-19 were exposed to greater functional dependence in their daily living activities. This study aims to examine the prevalence of functional dependence and associated factors among Brazilian older people with functional dependence 12 months after COVID-19 infection. **Methods:** A cross-sectional study was carried out involving people aged 60 years or older in the state of Paraná, Brazil. One year after notification or hospital discharge due to COVID-19, between June 2021 and March 2022, participants responded to a questionnaire via telephone call about sociodemographic data and data on functionality using the Measure of Functional Independence (FIM). The outcome variable “assessment of functional capacity” was divided into functional dependence (FIM Total < 104) and functional independence (FIM Total ≥ 104). **Results:** A total of 768 older adults participated, with an average age of 68.03 ± 6.8 years (range between 60 and 100). A majority of them were female (50.3%), white (46%), with low education (37.4%), had a partner (56.3%), did not live alone (72.4%), and had their own home (52.2%). The prevalence of functional dependence was 7.2%. On average, participants scored 5.4 points lower on FIM one year after COVID-19 infection compared with those in the acute phase of COVID-19 (125.5 vs. 120.1; *p* < 0.001). Functional dependence was higher (*p* < 0.05) among women when compared to men (aOR = 2.28); in people who changed their work situation due to COVID-19 when compared to those with no change (aOR = 5.27); in people with fair/poor/bad self-reported health compared to those with excellent/good health (aOR = 2.97); in people with cardiovascular symptoms compared to those without cardiovascular symptoms (aOR = 3.37); and among the most severe cases of the disease (treatment in ICU) compared to mild cases (outpatient treatment) (aOR = 10.5). **Conclusions:** Most participants presented functional independence 12 months after COVID-19 infection. Cases of functional dependence were influenced by multidimensional factors, including physical health, economic, and psychosocial aspects.

## 1. Introduction

COVID-19 disease has been a global pandemic since March 2020 [1]. Brazil has been one of the most affected countries in the world in terms of the absolute number of diagnosed cases, with more than 39 million cases and 714 thousand deaths due to COVID-19 reported to date [2]. People over 60 years represent 65.9% of cases of severe acute respiratory syndrome (SARS) hospitalized for COVID-19 in 2022, indicating that the risk of hospitalization and death remain higher among those aged over 60 [3].

COVID-19 is a multi-organ disorder and, in addition to respiratory symptoms, several other symptoms develop, including cardiovascular, gastrointestinal, neurological, and musculoskeletal complications [4]. Furthermore, it is estimated that more than 142 million older people, or 14% of all people aged 60 or older worldwide, currently fail to meet all their basic daily needs [5]. The impact of COVID-19 on functional capacity was observed in patients with mild COVID-19, as well as hospitalized patients with moderate-to-severe cases who required respiratory support or intensive care [6,7].

This study adopted the World Health Organization’s concept of functional capacity, conceived as an interaction between an individual’s physical and mental resources and their environment when conducting activities considered important for themselves and for their survival [5]. The expanded conception of health and aging considers functional capacity as an indicator of physical, mental, and social well-being [8]. In this vein, Rahmati et al. [9] underscore physical activity’s potential as a preventative measure against mental health complications, highlighting the importance of promoting physical activity in mental health interventions.

Recent research involving the general population during COVID-19 has shown that people have persistent symptoms regardless of whether they have certain symptoms (or groups of symptoms) or are hospitalized during the acute phase of the disease [10]. Previous studies identified factors associated with the decline in functional capacity after COVID-19: pre-admission frailty, stroke, history of depressive disorder, complications, length of hospitalization, and age, thereby demonstrating a complex network of multidimensional factors related to functionality [11,12,13]. Basic daily activities before COVID-19, female gender, and elevated plasma levels of D-dimer on admission were associated with a higher risk of functional decline [14].

The demand for post-acute rehabilitation following COVID-19 is rising, particularly among older individuals with preexisting health conditions [15]. Medical rehabilitation is an essential element in the ongoing care of patients. It aids in mitigating or decelerating the deconditioning effects, including the debilitating consequences of immobilization, joint stiffness, and pain, which often co-occur, particularly in older patients. Hence, rehabilitation enhances long-term recovery and functional autonomy [16,17]. Rehabilitation programs must be customized to address each patient’s specific needs, targeting the diverse deficits resulting from COVID-19, as well as pre-existing diseases and/or frailty [15]. Every patient requires a tailored rehabilitation program that encompasses “breathlessness control, strength training, functional and vocational rehabilitation, aerobic exercise, balance training, and psychological support” [18] (p. 1).

Considering the reduction of homeostatic reserve in the aging process and the greater chance of irreversibility when functional losses occur [19], it is important to identify risk factors that may cause or aggravate the decline in older people’s functional capacity, compromising their autonomy and/or independence for the performance of Daily Living Activities (DLAs) and, therefore, their quality of life [20]. While the literature does provide data on the association between functional status and clinical profile of older people with COVID-19 [21,22], the prognostic value of functional dependence in relation to sociodemographic factors, health, and treatment of COVID-19 is scarce. The understanding of these factors should translate to an individualized care plan to increase autonomy and reduce dependence. Thus, it is extremely important to analyze the factors associated with functional dependence, since the impairment of motor and cognitive functions impairs daily living activities, compromises labor activities, and significantly impacts the individual’s quality of life.

Based on the hypothesis that diseases such as COVID-19 can change the functional capacity of older adults, the study aimed to examine the prevalence of functional dependence and associated factors among older people with functional dependence 12 months after COVID-19 infection.

## 2. Materials and Methods

### 2.1. Study Design

This is an observational cross-sectional study involving older people who lived in the community and recovered from COVID-19 infection in the period between March and December 2020. This study is part of an ambispective cohort study investigating longitudinal follow-up of people who had COVID-19 in the state of Paraná [23].

This study follows the Strengthening the Reporting of Observational Studies in Epidemiology (STROBE) statement for the reporting of observational studies [24]. 

### 2.2. Sample and Recruitment

The study population was recruited through health surveillance systems called the Influenza Epidemiological Surveillance Information System (SIVEP-Influenza) and COVID-19 Paraná Notification System. To calculate the required sample size, we considered proportional stratified sampling according to the month of notification/hospital discharge and the macro-region of residence. A 95% confidence level and 80% statistical power were defined to detect a relative risk ratio of at least 1.75 in older people with COVID-19 for a functional dependence prevalence of at least 10% [25]. Hence, at least 712 participants were needed for the study.

Participants in this study were stratified based on treatment site, which was used as a determinant of disease severity. Participants who followed up in an outpatient clinic were considered mild cases, those hospitalized in the medical ward were moderate cases, and those admitted to the Intensive Care Unit (ICU) were severe cases. The study included old adults aged 60 or older, with a confirmed COVID-19 diagnosis; who were living in the state of Paraná, Brazil; who, during the disease’s acute phase, received treatment for COVID-19 in an outpatient clinic, medical ward, or ICU; and who were able to give their informed consent. In cases of communication inability at the time of contact, the responsible family caregiver filled out the form.

Overall, 900 older adults were assessed for eligibility, but 132 were excluded: 7 participants died, 53 did not complete the functional-capacity assessment form completely, and 72 presented functional dependence before COVID-19. Finally, the data of 768 older people were analyzed.

### 2.3. Data Collection

Data were collected via a telephone call between June 2021 and March 2022. Participants were interviewed 12 months after notification (for COVID-19 mild cases) or hospital discharge (for moderate and severe cases of COVID-19). The study period represents patients whose symptom onset was before 7 June 2021, when >75% of patients had an infection with the Alpha variant [26].

The following instruments were used:

(1) Sociodemographic, health, and treatment data (independent variables)—sex (male; female); age group (60–74 (young–old); ≥75 (old–old); race (white/non-white); years of study (up to 8; more than 8); has a partner (no/yes); lives alone (no/yes); housing condition (own; rented/other); estimated per capita family income (up to 1 minimum wage; more than 1 minimum wage); changed work situation (no; yes, due to COVID-19; yes, other reasons); change in income due to COVID-19 (no/yes); received financial aid or assistance from the government (no/yes); physical activity (up to 150 min per week; more than 150 min per week); alcohol use (no/yes); tobacco use (no/yes); self-perceived health (excellent/good; fair/poor/terrible); self-perception of quality of life (excellent/good; fair/poor/terrible); polypathology (≥5 diseases) (no/yes); long-term use of medication (no/yes); overweight/obesity (BMI “body weight (kg)/height (m2)” ≥ 27 kg/m^2^) (no/yes); symptoms in acute phase of COVID-19 (neurological, respiratory, digestive, endocrine, skin, musculoskeletal, cardiovascular, and psychosocial) (no/yes); treatment location (outpatient; medical ward; intensive care unit); use of ventilatory support (no/yes); cost of treatment through SUS (public healthcare system) (no/yes).

(2) Functional Independence Measure (FIM; outcome variable): The Brazilian Portuguese version of FIM was translated and validated by Riberto [27]. This instrument evaluates quantitatively the burden of care required by a person to perform a series of instrumental motor and cognitive tasks of daily living. It evaluates 18 items, 13 of which are motor (including 6 self-care items, 2 sphincter control items, 3 transfer items, and 2 motor items), and 5 cognitive (including 2 communication items and 3 social cognition items). Each item has a maximum score of 7 points and a minimum score of 1 point. The total FIM ranges “from 18 to 126, and can be divided into four categories, according to the total score obtained: (a) 18 points: complete dependence (total assistance); (b) 19–60 points: modified dependence 1 (assistance with up to 50% of tasks); (c) 61–103 points: modified dependence 2 (assistance with up to 25% of tasks); (d) 104–126 points: complete/modified independence” [27] (p. 1098). Domains can also be analyzed separately. In this case, motor FIM varies from 13 to 91, and cognitive FIM from 5 to 35. Therefore, higher scores indicate better functional performance [27,28]. The internal consistency of FIM and its subscales was excellent. This instrument was used in previous studies on functional status and COVID-19 [21,29].

A cut-off value for the FIM (global score < 104) was used to stratify dependent and independent performance in daily-living activities, as in previous research [30]. Functional dependence (FIM Total < 104) or independence (FIM Total ≥ 104) was considered the outcome variable to evaluate factors associated with the functional capacity of the older person.

### 2.4. Statistical Analysis

The data were compiled in electronic spreadsheets. Subsequently, a descriptive analysis (absolute and relative frequencies) was performed for the sociodemographic, health, and treatment variables. The association between these variables and functional dependence was verified by Pearson’s chi-squared test (5% significance level).

Univariate and multivariate logistic regression models were employed to find the potential explanatory variables for the functional dependence of older adults affected by COVID-19. The variables that yielded a *p*-value below 20% (*p* < 0.20) in the bivariate analysis were incorporated individually into the multivariate model. The stepwise regression analysis was employed to pick variables and refine the final models. The goodness fit of these models was assessed through an examination of random quantile residuals, the Hosmer–Lemeshow test, the area under the ROC curve (AUC), and McFadden’s pseudo-R^2^; collinearity was evaluated using the variance inflation factor (VIF). Associations were estimated by calculating the Odds Ratio (OR), adopting the 95% confidence interval as a measure of accuracy [31]. After excluding all missing data, analyses were performed using Software R version 4.3.2.

### 2.5. Ethics

The study was conducted after approval of the Permanent Committee of Ethics in Research involving Human Beings (COPEP) of the State University of Maringá (UEM), with favorable opinion number 4,165,272/2020. Participants provided verbal consent before conducting the interviews and this acceptance was recorded. This study also complies with Resolutions 466/2012 and 510/2016 of the National Health Council.

## 3. Results

### 3.1. Sample Description

Of the 768 participants included in this study, 50.3% (n = 386) were female (Table 1). The prevalent age group was young–old (60 to 74), corresponding to 82.4% (n = 633), with an average of 68.03 ± 6.8 years (range between 60 to 100). Regarding ethnicity, 46% (n = 353) declared themselves white. Most participants had up to 8 years of study (37.4%; n = 287), had a partner (56.3%; n = 432), did not live alone (72.4%; n = 556), and had their own housing (52.2%; n = 401); and their per capita family income was above 1 minimum wage (14.1%; n = 108). Regarding the social repercussions of COVID-19, 64.6% (n = 496) of participants reported no change in their work situation, no change in income due to COVID-19 (55.8%; n = 429), and not receiving financial aid or government aid (57.3%; n = 440).

Regarding health variables, around 23.2% (n = 178) of participants practiced more than 150 min of physical activity per week, 54.3% (n = 417) did not consume alcohol, and 24.7% (n = 190) were smokers. Most participants reported their health and quality of life as excellent or good, while 40.3% (n = 310) were overweight or obese, and 64.3% had at least one comorbidity. Cardiovascular (n = 438; 52.1%) and endocrine (n = 228; 27.1%) diseases were the most prevalent.

The most common symptom in the acute phase of COVID-19 was neurological (66.1%; n = 508), followed by respiratory (56.5%; n = 434) and digestive (47.1%; n = 362). A significant proportion required hospitalization (57.6%; n = 442), used ventilatory support (39.7%; n = 305), and received invasive mechanical ventilation (9.9%; n = 69). Treatment costs supported by the public healthcare system (SUS) were present in 53.4% of cases (n = 410).

### 3.2. Prevalence of Functional Dependence Among Study Participants 12 Months After COVID-19 Infection

One year after COVID-19 infection and based on FIM, 55 participants (54.5%) were assigned to the functional dependent group (prevalence = 7.2%), and the remaining 713 were assigned to the functional independent group. The total FIM mean was 120.1 ± 17.1. On average, participants scored 5.4 points lower on FIM one year after COVID-19 infection compared with those in the acute phase of COVID-19 (125.5 vs. 120.1; *p* < 0.001).

Table 2 shows the association of total FIM with the other study variables. There were significant associations between functional dependence and female sex (*p* = 0.006); altered work situation due to COVID-19 (*p* < 0.001); self-perception of health (*p* < 0.001); quality of life (*p* = 0.001); polypathologies (*p* < 0.001); long-term use of medication (*p* = 0.010); treatment site (*p* < 0.001); use of ventilatory support (*p* < 0.001); and, finally, presence of symptoms in the acute phase of COVID-19, such as neurological (*p* = 0.016), respiratory (*p* = 0.036), digestive (*p* = 0.016), endocrine (*p* = 0.033), musculoskeletal (*p* = 0.0059), cardiovascular (*p* < 0.001), and psychosocial (*p* = 0.036).

### 3.3. Factors Associated with Functional Dependence

After adjusting for factors associated with functional dependence, the effect of the variables sex, age group, altered work situation, self-perception of health, cardiovascular symptoms, and treatment location was maintained in the multiple analyses. The factors associated with functional dependence in post-COVID-19 were female (aOR = 2.28), age 75 or older compared to age group 60–74 (aOR = 2.87), reporting a changed work situation due to COVID-19 (aOR = 5.27), self-perceived health (aOR = 2.97), cardiovascular symptoms in the disease’s acute phase (aOR = 3.37), and hospitalization in ICU (aOR = 10.5) (Table 3). Being male, being aged between 60 and 74, having no altered work situation, having self-reported health as excellent/good, not having symptoms with cardiovascular symptoms, and having received outpatient treatment were protective factors. The normality test for random quantile residuals yielded *p* = 0.71, the Hosmer–Lemeshow test resulted in *p* = 0.14, and the AUC was 0.86. The model was able to explain 36.8% of the total variance in functional dependence (McFadden’s pseudo-R^2^ = 0.368; *p* < 0.001).

## 4. Discussion

This study found that functional dependence was present in 7.2% of participants with confirmed SARS-CoV-2 infection 12 months after notification or hospital discharge in the state of Paraná, Brazil. Functional dependence was associated with gender, age, altered work status due to COVID-19, negative self-perception of health, and cardiovascular symptoms in the disease’s acute phase and severity in the acute phase of COVID-19 (hospitalization in ICU). Among these factors, greater disease severity and changes in the work situation due to COVID-19 were the strongest predictors.

Being female was identified as a risk factor for functional dependence when compared to males. Females were significantly associated with functional dependence in other studies [32,33,34]. Health-seeking behavior in men is lower than in women, and men may be less inclined to report the persistence of health problems [35]. However, other factors, such as the effect of sex hormones on the risk of developing post-COVID-19 syndrome, socioeconomic effects of COVID-19, and disparities in access to health, need to be further explored.

Overall, older people have a higher risk of developing functional dependence after an acute condition [36], and this study showed a significant association between advanced age and functional dependence. Similar results were also reported in previous studies [21,34]. Older adults are particularly affected by COVID-19, and older age can make the diagnosis more complex. Lung infections in older people are often difficult to treat and result in a higher mortality rate than in younger people. Generalized deterioration, weakness, abdominal symptoms, anorexia, confusion, tachycardia, and tachypnea may signal the onset of pneumonia. The diagnosis of pneumonia may go unnoticed since the classic symptoms of cough, chest pain, sputum production, and fever may be absent or masked in older patients [37]. Thus, during the care of older people due to COVID-19, the presence and impact of this factor should be considered, especially in more advanced ages.

Instrumental activities of daily living (IADLs) are primarily affected in the case of disability [38,39]. COVID-19 has significantly impacted the lives of older people, who changed their work situation after contracting the disease, a factor that impairs activities of daily living and significantly impacts the individual’s quality of life.

Admission to the ICU was significantly associated with worse functional capacity. Hospitalization for COVID-19 can cause persistent functional consequences after hospital discharge due to the direct and indirect effects of SARS-CoV-2 on various organs and systems, in addition to post-intensive care syndrome and prolonged bed rest [40]. In addition, the lack of knowledge of the particularities of the aging process can generate interventions capable of worsening the health of the elderly, known as iatrogenic disease. Iatrogenesis reflects the harm caused by health professionals and health systems unprepared to provide an adequate response to the health problems of the elderly [25].

Polypathology seems to negatively impact older people, contributing to an increased risk of functional dependence. The presence of comorbidities and aging represents important risk factors for disease severity and poor prognosis [41]. Despite this, people of any age with severe underlying medical conditions are more at risk of contracting COVID-19 infection. Older people infected with SARS-CoV-2 and with comorbidities, including Parkinson’s disease, diabetes, cancer, hypertension, and cardiovascular diseases, are at increased risk of death [42]. In this study, cardiovascular symptoms were associated with older people’s functional dependence in the post-COVID-19 period. A systematic review evaluated cardiac sequelae after recovery from COVID-19, and indicated that it appears to be associated with persistent cardiac injury after recovery, particularly subclinical myocardial injury in the earlier phase and diastolic dysfunction later [43]. A current hypothesis about the effects of long COVID-19 on the functional state is that patients continue to have an abnormal immune system due to remnants of a continuous and sustained inflammatory process, even after the viral infections acute phase. The reasons for this activated inflammation require further investigation, but possibilities include antigen persistence, autoimmune responses by antigenic cross-reactivity, or a damaged repair reflex [44].

The results showed a high level of functional independence among the studied sample (92.8%). In a similar study, Battistella et al. [45] reported slightly lower functional independence (86.53%) in a sample of 800 Brazilians with an average age of 55.35, demonstrating that older people infected with COVID-19 have long-term improvements in DLAs. This apparent contradiction can be explained by the higher proportion of severe cases compared to this study. Whereas 75% of people’s intrinsic capacity, namely physical and mental resources, is largely due to the accumulated effects of people’s habits and the environmental factors to which they have been exposed, there is a strong impact of social determinants on the functioning of an older person [5]. In parallel, when we compared data from the pre- and post-pandemic (2009–2021), there was also a decrease in aerobic and muscle-strengthening activities in young people, which reinforces the need to encourage physical activities through public health policies [46].

When the treatment site is compared with functional capacity, it is observed that hospitalization in ICU culminated in decreased functionality of the participants. The same trend is apparent in other studies, including that of Teixeira-Vaz [47], who conducted a Portuguese cohort study including 42 patients who had COVID-19 and were treated in the ICU between May 2020 and September 2021. This study revealed that, 12 months after hospital discharge, 26% of patients were not fully independent [47]. These results show the importance of a multidisciplinary team in planning and implementing care for hospitalized older patients in order to mitigate negative repercussions, such as deterioration of functional capacity. The relationship with disease severity indicates a direct role of infection in the development of functional dependence, in addition to general factors related to hospitalization. Other short- and long-term neuropsychiatric sequelae after SARS-CoV-2 infection and autoimmune inflammatory rheumatic diseases were found in different ethnic cohorts [48,49].

Negative self-perception of quality of life was associated with functional dependence even 12 months after notification or discharge. This is in line with a multicenter study that reported lower quality of life following a COVID-19 infection [50]. Social distancing and hospitalization can affect the daily activity and physical functioning of the elderly, adversely affecting their quality of life [51]. Other studies with patients who recovered from COVID-19 and were followed up to one year after hospitalization revealed that the number of comorbidities significantly influenced health-related quality-of-life trajectories [52,53]. In addition to comorbidities, race, home ownership, daily screen time, musculoskeletal and anxiety symptoms, and work situation were significant predictors of quality of life among older COVID-19 survivors [54]. Although the results of the present study did not show a statistically significant relationship between the practice of physical activity and functional dependence, evidence points to the potential of physical activity as a preventative measure against mental health complications, highlighting the importance of promoting physical activity in mental health interventions [9].

The present study had several limitations. First, the self-reported data we used are from a single region of Brazil (Paraná), and missing data may have affected the results. Second, the study included a relatively small cohort of patients. Extrapolation of data from one area of Brazil to other countries is not possible, given the great variability of functional status in older people. Further research should evaluate larger samples from other regions or countries with different healthcare systems and populations. In addition, further elucidation of the principal causal elements of functional impairments post-COVID-19 is required. Health education campaigns addressing the health ramifications of post-COVID-19 are necessary. Third, the absence of a control group hindered the ability to determine whether the self-reported symptoms were attributable to SARS-CoV-2 infection, previous comorbidities, or social consequences connected to the pandemic. The ability to make a distinction between COVID-specific effects and general aging effects is hindered by the absence of a control group (non-COVID-infected older adults). Fourth, the need to conduct telephone interviews regarding social-distancing measures limited the participation of older people due to (1) social vulnerability and access to the telephone; and (2) distrust in telephone calls given the possibility of scams, telemarketing, etc. Fifth, most of the studies published so far have followed hospitalized COVID-19 patients, limiting the discussion of mild and moderate cases. Lastly, all participants were first diagnosed with COVID-19 before June 2021, when either wild-type or the alpha variant were prominent and before the wide dissemination of COVID-19 vaccines [26]. This sample feature reduces the generalizability of findings when applied to more recent variants. Despite these limitations, the use of validated scales applicable in clinical practice confers greater consistency to the results obtained. This study also encompassed a substantial cohort of individuals who did not need hospitalization, contrasting with most studies that focus on the functional condition of critical patients.

## 5. Conclusions

Regarding the evaluation of functional status, most participants were classified by FIM as independent in their activities of daily living; however, the frailty among the older adults 12 months after infection was considerable. Thus, they would benefit from the early adoption of functional and psychosocial rehabilitation measures to help affected older people develop adaptive strategies, rearrange their routines, and access specialized health services whenever needed [55,56].

Although the most critical period of the COVID-19 pandemic has passed, the long-term consequences of SARS-CoV-2 infection remain a relevant concern, especially for vulnerable groups such as older people. Many post-COVID-19 conditions, including functional and cognitive impacts, are still being investigated, as they may affect quality of life, public health, and health systems in the coming years. Studies such as this one expand the understanding of mild and moderate cases, which have been underexplored in the literature predominantly focused on hospitalized patients. In addition, in the coming years, global health systems will still be dealing with the late and indirect effects of the pandemic, such as the worsening of chronic diseases, high old-age dependency ratios, overload of rehabilitation services, and rising social security costs. The relevance of this study lies not only in providing unprecedented data on specific populations but also in contributing to management strategies, public policies, and health education that help mitigate the persistent impacts of COVID-19.

The present study found an association between functional dependence and the female gender, advanced age, presence of cardiovascular symptoms in the acute phase of COVID-19, greater disease severity, and participants’ social life. This contributes to a negative self-perception of health. In addition, changes in the work situation harmed functional capacity, increasing the social vulnerability of participants. These findings highlight the importance of evaluating and preventing functional dependence post-COVID-19, which depends on multiple conditional factors, such as physical, social, clinical, and treatment. Therefore, early identification of older people with risk factors for functional dependence is necessary to support their complete care. In addition, extended monitoring is necessary to determine whether these functional limitations after recovery from COVID-19 persist or not.

## Figures and Tables

**Table 1:** Description of sample characteristics (N = 768).

Variables	Frequency n	Percentage %
**Sociodemographic**		
Sex		
Male	382	49.7
Female	386	50.3
Age range (years)		
60 to 74 (young–old)	633	82.4
≥75 (old–old)	135	17.6
Race/Color		
White	353	46.0
Non-white	157	20.4
No information	258	33.6
Years of study		
Up to 8	287	37.4
More than 8	203	26.4
No information	278	36.2
Has a partner		
No	177	23.0
Yes	432	56.3
No information	159	20.7
Lives alone		
No	556	72.4
Yes	103	13.4
No information	109	14.2
Housing condition		
Own	401	52.2
Rented/other	56	7.3
No information	311	40.5
Estimated per capita family income *		
Up to BRL 1100.00	108	14.1
More than BRL 1101.00	129	16.8
No information	531	69.1
Changed work situation		
No	496	64.6
Yes, due to COVID-19	24	3.1
Yes, other reasons	37	4.8
No information	211	27.5
Change in income due to COVID-19		
No	429	55.8
Yes	118	15.4
No information	221	28.8
Received financial aid or assistance from the government		
No	440	57.3
Yes	90	11.7
No information	238	31.0
**Health**		
Physical activity		
Up to 150 min per week	46	6.0
>150 min per week	178	23.2
No information	544	70.8
Alcohol use		
No	417	54.3
Yes	158	20.6
No information	193	25.1
Tobacco use		
No	144	18.8
Yes	190	24.7
No information	434	56.5
Self-perceived health		
Excellent/good	461	60.0
Fair/poor/terrible	137	17.8
No information	170	22.2
Self-perception of quality of life		
Excellent/good	486	63.3
Fair/poor/terrible	95	12.4
No information	187	24.3
Polypathology (≥5 diseases)		
No	728	94.8
Yes	40	5.2
Long-term use of medication		
No	398	51.8
Yes	187	24.4
No information	183	23.8
Overweight/obesity		
(BMI ≥ 27 kg/m^2^) §		
No	165	21.5
Yes	310	40.3
No information	293	38.2
**Symptoms in acute phase of COVID-19**		
Neurological symptoms		
No	260	33.9
Yes	508	66.1
Respiratory symptoms		
No	334	43.5
Yes	434	56.5
Digestive symptoms		
No	406	52.9
Yes	362	47.1
Endocrine symptoms		
No	586	76.3
Yes	182	23.7
Skin symptoms		
No	714	93.0
Yes	54	7.0
Musculoskeletal symptoms		
No	640	83.3
Yes	128	16.7
Cardiovascular symptoms		
No	727	94.7
Yes	41	5.3
Psychosocial symptoms		
No	498	64.8
Yes	270	35.2
**Treatment**		
Treatment location		
Outpatient	326	42.4
Medical ward	231	30.1
Intensive Care Unit (ICU)	211	27.5
Use of ventilatory support		
No	381	49.6
Yes	305	39.7
No information	82	10.7
Cost of treatment through SUS ¥		
No	358	46.6
Yes	410	53.4

* The minimum wage value in Brazil is BRL 1100.00 for the year 2020; §, Body Mass Index; ¥, SUS, public healthcare system.

**Table 2:** Sociodemographic, health, and treatment variables according to functional dependence.

Independent Variables	Functional Dependency (FIM Global Score < 104)				
	Yes		No		
	**n**	**%**	**n**	**%**	*p* **
**Sociodemographic**					
Sex					
Male	17	30.9	365	51.2	**0.006**
Female	38	69.1	348	48.8	
Age range (years)					
60 to 74 (young–old)	41	74.5	592	83.0	0.159
≥75 (old–old)	14	25.5	121	17.0	
Race/Color					
White	36	78.3	317	68.3	0.220
Non-white	10	21.7	147	31.7	
Years of study					
Up to 8	22	48.9	265	59.6	0.221
More than 8	23	51.1	180	40.4	
Has a partner					
No	17	34.0	160	28.6	0.522
Yes	33	66.0	399	71.4	
Lives alone					
No	45	86.5	511	84.2	0.803
Yes	7	13.5	96	15.8	
Housing condition					
Own	41	91.1	360	87.4	0.627
Rented/other	4	8.9	52	12.6	
Estimated per capita family income *					
Up to BRL 1100.00	16	48.5	92	45.1	0.862
More than BRL 1101.00	17	51.5	112	54.9	
Changed work situation					
No	35	71.4	461	90.7	**<0.001**
Yes, due to COVID-19	9	18.4	15	3.0	
Yes, other reasons	5	10.2	32	6.3	
Change in income due to COVID-19					
No	34	69.4	395	79.3	0.153
Yes	15	30.6	103	20.7	
Received financial aid or assistance from the government					
No	32	76.2	408	83.6	0.311
Yes	10	23.8	80	16.4	
**Health**					
Physical activity					
Up to 150 min per week	4	21.1	42	20.5	0.978
>150 min per week	15	78.9	163	79.5	
Alcohol use					
No	36	75.0	381	72.3	0.816
Yes	12	25.0	146	27.7	
Tobacco use					
No	35	72.9	399	75.3	0.850
Yes	13	27.1	131	24.7	
Self-perceived health					
Excellent/good	25	49.0	436	79.7	**<0.001**
Fair/poor/terrible	26	51.0	111	20.3	
Self-perception of quality of life					
Excellent/good	33	64.7	453	85.5	**0.001**
Fair/poor/terrible	18	35.3	77	14.5	
Polypathology (≥5 diseases)					
No	45	81.8	683	95.8	**<0.001**
Yes	10	18.2	30	4.2	
Long-term use of medication					
No	23	50.0	375	69.6	**0.010**
Yes	23	50.0	164	30.4	
Overweight/Obesity					
No	10	22.2	155	36.0	0.091
Yes	35	77.8	275	64.0	
**Symptoms in acute phase of COVID-19**					
Neurological symptoms					
No	10	18.2	250	35.1	**0.016**
Yes	45	81.8	463	64.9	
Respiratory symptoms					
No	16	29.1	318	44.6	**0.036**
Yes	39	70.9	395	55.4	
Digestive symptoms					
No	20	36.4	386	54.1	**0.016**
Yes	35	63.6	327	45.9	
Endocrine symptoms					
No	35	63.6	551	77.3	**0.033**
Yes	20	36.4	162	22.7	
Skin symptoms					
No	48	87.3	666	93.4	0.150
Yes	7	12.7	47	6.6	
Musculoskeletal symptoms					
No	38	69.1	602	84.4	**0.006**
Yes	17	30.9	111	15.6	
Cardiovascular symptoms					
No	40	72.7	687	96.4	**<0.001**
Yes	15	27.3	26	3.6	
Psychosocial symptoms					
No	28	50.9	470	65.9	**0.036**
Yes	27	49.1	243	34.1	
**Treatment**					
Treatment location					
Outpatient	5	9.1	321	45.0	**<0.001**
Medical ward	11	20.0	220	30.9	
ICU	39	70.9	172	24.1	
Use of ventilatory support					
No	9	17.0	372	58.8	**<0.001**
Yes	44	83.0	261	41.2	
Cost of treatment through SUS					
No	29	52.7	329	46.1	0.422
Yes	26	47.3	384	53.9	

* The minimum wage value in Brazil is BRL 1100.00 for the year 2020; ** Pearson’s chi-squared test; BMI, Body Mass Index; ICU, Intensive Care Unit; bold means *p* < 0.05.

**Table 3:** The adjusted odds of functional dependence among older adults 12 months after SARS-CoV-2 infection.

Variables	Estimate (Beta)	aOR (CI 95%)	*p*
Sex			
Male	Reference	-	
Female	0.82	2.28 (1.02;5.1)	**0.044**
Age range (years)			
60 to 74 (young–old)	Reference	-	
≥75 years (old–old)	1.05	2.87 (1.15;7.12)	**0.023**
Work situation			
Not changed	Reference	-	
Changed due to COVID-19	1.66	5.27 (1.63;17.09)	**0.006**
Self-perceived health			
Excellent/good	Reference	-	
Fair/bad/terrible	1.09	2.97 (1.37;6.41)	**0.006**
Cardiovascular symptoms			
No	Reference	-	
Yes	1.21	3.37 (1.33;8.52)	**0.010**
Treatment location			
Outpatient	Reference	-	
ICU	2.35	10.5 (3.36;32.82)	**0.001**

aOR, adjusted Odds Ratio; CI, confidence interval; bold means *p* < 0.05.

## Data Availability

The raw data supporting the conclusions of this article will be made available by the authors upon request.
